# Adenylyl Cyclase in Ocular Health and Disease: A Comprehensive Review

**DOI:** 10.3390/biology13060445

**Published:** 2024-06-18

**Authors:** Polly Thompson, Virginija Vilkelyte, Malgorzata Woronkowicz, Mitra Tavakoli, Piotr Skopinski, Harry Roberts

**Affiliations:** 1 West of England Eye Unit, Royal Devon University Healthcare NHS Foundation Trust, Exeter EX2 5DW, UK; polly.thompson5@nhs.net; 2 University of Exeter Medical School, St Luke’s Campus, University of Exeter, Exeter EX1 2HZ, UK; 3 NDDH, Royal Devon University Healthcare NHS Foundation Trust, Barnstaple EX31 4JB, UK; malgorzata.woronkowicz.14@alumni.ucl.ac.uk; 4 Moorfields Eye Hospital NHS Foundation Trust, 162 City Road, London EC1V 2PD, UK; 5 Department of Ophthalmology, SPKSO Ophthalmic University Hospital, Medical University of Warsaw, 00-576 Warsaw, Poland; 6 Department of Histology and Embryology, Medical University of Warsaw, 02-004 Warsaw, Poland

**Keywords:** adenylyl cyclase, soluble adenylyl cyclase, cornea, retina, retinal ganglion cells, lacrimal gland, biomarker, dry eye disease

## Abstract

**Simple Summary:**

Adenylyl cyclase is an enzyme that plays an important role in cellular signalling in mammals. This review gathers current understanding of the role of adenylyl cyclase in the eye, covering the cornea, lens, retina, lacrimal gland and tear film, and explores their potential as targets for therapeutic innovation.

**Abstract:**

Adenylyl cyclases (ACs) are a group of enzymes that convert adenosine-5′-triphosphate (ATP) to cyclic adenosine 3′,5′ monophosphate (cAMP), a vital and ubiquitous signalling molecule in cellular responses to hormones and neurotransmitters. There are nine transmembrane (tmAC) forms, which have been widely studied; however, the tenth, soluble AC (sAC) is less extensively characterised. The eye is one of the most metabolically active sites in the body, where sAC has been found in abundance, making it a target for novel therapeutics and biomarking. In the cornea, AC plays a role in endothelial cell function, which is vital in maintaining stromal dehydration, and therefore, clarity. In the retina, AC has been implicated in axon cell growth and survival. As these cells are irreversibly damaged in glaucoma and injury, this molecule may provide focus for future therapies. Another potential area for glaucoma management is the source of aqueous humour production, the ciliary body, where AC has also been identified. Furthering the understanding of lacrimal gland function is vital in managing dry eye disease, a common and debilitating condition. sAC has been linked to tear production and could serve as a therapeutic target. Overall, ACs are an exciting area of study in ocular health, offering multiple avenues for future medical therapies and diagnostics. This review paper explores the diverse roles of ACs in the eye and their potential as targets for innovative treatments.

## 1. Introduction

Adenylyl cyclases (ACs) are a group of enzymes that catalyse the conversion of adenosine-5′-triphosphate (ATP) to cyclic adenosine 3′,5′ monophosphate (cAMP) [1], a vital intermediate molecule in many signal transduction pathways, including cellular responses to hormones and neurotransmitters. AC originates from a variety of genes and has a regulatory role in nearly all cells [2]. 

Many structurally diverse inhibitors of AC have been identified. With regard to tmAC, they are divided into four classes: inhibitors that compete with the ATP at the catalytic site; non-competitive inhibitors which mimic the cAMP-transition state; allosteric non-competitive inhibitors which target the diterpene site; and allosteric non-competitive inhibitors which target currently undefined sites [3].

In humans, there are 10 unique AC genes [1]. One of the genes is soluble adenylyl cyclase (sAC), which is the most evolutionarily conserved, but the most recently identified [4]. The other nine genes encode transmembrane adenylyl cyclases (tmAC), which have been suggested to interact G-protein-coupled receptors (GPCRs) [3,4,5,6,7], proteins that detect molecules outside the cell and trigger an intracellular response.

sAC requires a divalent cation (such as Ca^2+^) and bicarbonate for activation [8,9,10,11,12,13,14]. It is expressed in many different cell types and can be found anywhere in the cell. sAC is not a transmembrane protein, nor is it regulated by G proteins. It, instead, interacts with bicarbonate and triggers local cAMP signalling, commonly found in metabolically active tissue. Many such tissues exist within the eye, implicating sAC as a target of study.

The metabolic needs of the various anatomical structure in the eye are wide-ranging. Bicarbonate-stimulated sAC plays a role in regulating the conventional outflow of aqueous in the ciliary body [4,8,15,16,17,18,19,20,21,22]. AC signalling pathways have been identified in animal models of intrinsic eye muscle kinetics, namely iris sphincter and ciliary body contraction through studies of urocortin 2, a peptide related to corticotropin-releasing factor [23,24,25]. 

In genetic studies in mice, it has been shown that sAC is necessary for retinal ganglion cell survival and axon growth [18,26]. It is required for the development of amacrine cells and, to a lesser degree, photoreceptors [27]. Choroidal tissue is an area of significant adenylyl (and guanylyl) cyclase second messenger activity, interacting with hormones and neurotransmitters that impact relaxation [28]. AC activation by forskolin in guineapig sclera has been shown to result in myopic shift accompanied by reduced collagen mRNA levels, implicating AC in the development and treatment of short-sightedness [29].

These commonly found and important molecules show promise as focuses for clinical treatments and preventative medicine. The presence of AC in the ciliary body provides a target for glaucoma and intraocular pressure-modulating treatments. There is also a link to lacrimal gland duct fluid secretion found in the study of cystic fibrosis transmembrane conductance regulator (CFTR) knockout mice [30]. Dopamine receptor activation modulates the circadian timing of AC signalling in mouse retina, as AC is the primary enzyme controlling cAMP in dark adapted photoreceptors [31]. 

The identification of adenylyl cyclases (ACs) in the eye and their anatomical location in the eye presents an important area of study, promising to enhance our physiological understanding and spur the development of new treatments for ophthalmological and other medical conditions. This review summarizes the current knowledge of ACs, with a particular focus on the less-studied soluble adenylyl cyclase (sAC), and elucidates their roles within the eye.

Adenylyl cyclases (ACs) are a group of enzymes that catalyse the conversion of adenosine-5′-triphosphate (ATP) to cyclic adenosine 3′,5′ monophosphate (cAMP) [1], a vital intermediate molecule in many signal transduction pathways, including cellular responses to hormones and neurotransmitters. AC has a regulatory role in nearly all cells, catalysing the same reaction but originating from unrelated gene families [2]. 

In humans, there are 10 unique AC genes [1]. One of the genes is soluble adenylyl cyclase (sAC), which is the most evolutionarily conserved but the most recently identified [3]. The other nine genes encode transmembrane adenylyl cyclases (tmAC), which interact physically with G-protein-coupled receptors (GPCRs) [4,5,6], proteins that detect molecules outside the cell and trigger an intracellular response.

sAC requires a divalent cation (such as Ca^2+^) and bicarbonate for activation [7,8,9,10,11,12,13]. It is expressed in many different cell types and can be found anywhere in the cell [4,7,14,15,16,17,18,19,20]. sAC is not a transmembrane protein, nor is it regulated by G proteins. It, instead, interacts with bicarbonate and triggers local cAMP signalling, commonly found in metabolically active tissue. Many such tissues exist within the eye, implicating sAC as a target of study.

The metabolic needs of the complex anatomical structure in the eye are wide-ranging. Bicarbonate-stimulated sAC plays a role in regulating the conventional outflow of aqueous in the ciliary body [21]. AC signalling pathways have been identified in the animal models of intrinsic eye muscle kinetics, namely iris sphincter and ciliary body contraction through studies of urocortin 2, a peptide related to corticotropin-releasing factor [22,23,24]. 

In genetic studies in mice, it has been shown that sAC is necessary for retinal ganglion cell survival and axon growth [16,25]. It is required for the development of amacrine cells and, to a lesser degree, photoreceptors [26]. Choroidal tissue is an area of significant adenylyl (and guanylyl) cyclase second messenger activity, interacting with hormones and neurotransmitters that impact relaxation [27]. AC activation by forskolin in guineapig sclera has been shown to result in myopic shift accompanied by reduced collagen mRNA levels, implicating AC in the development and treatment of short-sightedness [28].

These commonly found and important molecules show promise as focuses for clinical treatments and preventative medicine. The presence of AC in the ciliary body provides a target for glaucoma and intraocular pressure-modulating treatments. AC-associated proteins have been identified as potential biomarkers for Alzheimer’s disease in the proteomic analysis of tear fluid [29]. There is also a link to lacrimal gland duct fluid secretion found in the study of cystic fibrosis transmembrane conductance regulator (CFTR) knockout mice [30]. Dopamine receptor activation modulates the circadian timing of AC signalling in mouse retina, as AC is the primary enzyme controlling cAMP in dark adapted photoreceptors [31]. 

**Figure 1:**
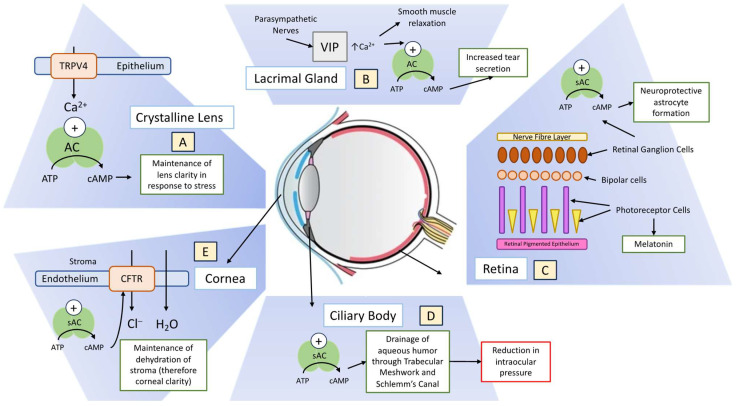
An overview of some of the functions of adenylyl cyclase (AC) and soluble adenylyl cyclase (sAC) in the eye. (**A**) The crystalline lens’s response to hypoosmotic stress involves the activation of transient receptor potential cation channel subfamily 4 (TRPV4), which maintains clarity through cAMP-mediated water and ion channel function. (**B**) Vasoactive peptide (VIP) released by parasympathetic nerves increases intracellular calcium. This causes smooth muscle cell relaxation and increased AC activity, which results in increased tear secretion. (**C**) Increased intracellular levels of cAMP catalysed by AC protects retinal ganglion cells (RGCs) from degeneration and promotes neuroprotective astrocyte formation. (**D**) cAMP is understood to facilitate the drainage of aqueous humour by the conventional outflow pathway, which is formed by the trabecular meshwork (TM) and Schlemm’s canal. (**E**) Increases in cAMP activity (modulated in places by the cystic fibrosis transmembrane conductance regulator (CFTR)) results in increased net endothelial fluid transport.

The identification of adenylyl cyclases (ACs) in the eye and their anatomical location in the eye present an important area of study, promising to enhance our physiological understanding and spur the development of new treatments for ophthalmological and other medical conditions. This review summarizes the current knowledge of ACs, with a particular focus on the less-studied soluble adenylyl cyclase (sAC), and elucidates their roles within the eye (Figure 1). 

## 2. Methodology

Our research was conducted using a combination of online databases, including PubMed, Google Scholar, and Web of Science. Keywords such as “adenylyl cyclase”, “AC”, “eye”, “ophthalmology”, and “ocular biomarkers” were employed to identify relevant articles. The search was conducted within a timeline spanning the past two decades to ensure the inclusion of recent developments. Additionally, manual searches of reference lists from relevant articles were performed to identify additional sources. This comprehensive search strategy aimed to gather a diverse range of studies and insights into the role of adenylyl cyclase in the eye.

### 2.1. Soluble Adenylyl Cylase

In 1975, a different type of adenylyl cyclase was found in mammals, distinct from the trans-membrane types which had been extensively studied [6]. A soluble source of AC activity was detected in the testis, with activity thought to be dependent on manganese and insensitive to G-protein and forskolin regulation. The physiological function, biochemical regulation and molecular nature did not become clear until the sAC protein was purified and cloned in 1999 [3]. sAC has since been identified abundantly in the cornea, ciliary body, retina and in epithelial cells lining the anterior surface of the lens [18,19,32,33,34].

The single functional sAC gene in the human genome (ADCY10) is composed of 33 exons and spans more than 100 kb, though it uses multiple promotors, with extensive alternative splicing [13]. Full-length mammalian sAC (sAC_fl_) comprises two heterologous catalytic domains (C1 and C2) which make up the 50 kDa amino terminus [13]. When compared to the minimal functional sAC variant (sAC_t_), the extra 140 kDa terminus of sAC_fl_ reflects several regulatory functions, such as a canonical P-loop, leucine zipper and autoinhibitory regions. sAC_t,_ on the other hand, is almost exclusively comprised of C1 and C2, making it several times stronger at forming cAMP [13]. 

The key amino acid residues implicated in the catalytic conversion of ATP to cAMP are conserved in cyanobacterial and mammalian sAC. ATP, with Ca^2+^ bound to its γ-phosphate, interacts with specific residues in the sAC catalytic centre, resulting in an ‘open sAC state’. Then, the second divalent metal ion binds the α-phosphate of ATP, leading to a ‘closed state’ [35]. The change from ‘open’ to ‘closed’ induces the esterification of the α-phosphate with the ribose in adenosine and the simultaneous release of the β- and γ- phosphates (‘cyclising’) [13]. 

**Figure 2:**
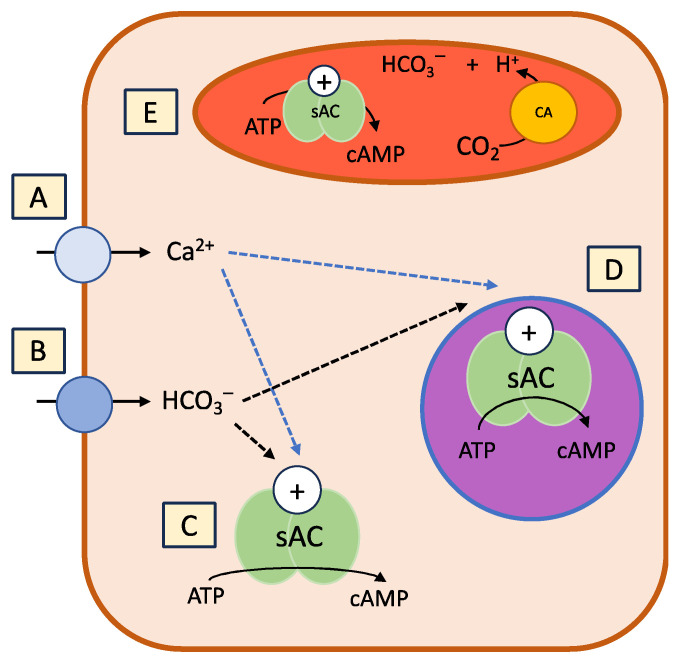
The activation of soluble adenylyl cyclase (sAC) by bicarbonate and Ca^2+^. (**A**) Ca^2+^ entering the cell through membrane transporters, or potentially from the release of the endoplasmic reticulum or mitochondria. (**B**) Bicarbonate entering through membrane-transporting proteins such as Cystic Fibrosis Transmembrane Conductance Regulators (CFTRs). (**C**) sAC activation in the cytoplasm. (**D**) Bicarbonate- and Ca^2+^-activating sAC in the nucleus. (**E**) sAC activated by metabolically generated CO_2_ through carbonic anhydrase (CA). Adapted from Tresguerres et al. [13].

The source of bicarbonate-regulating sAC can be metabolically generated or external to the cell (Figure 2). Carbonic anhydrases (CAs) found both intra- and extra-cellularly are essential for the fast hydration of CO_2_ into bicarbonate, which activates sAC [36]. 

### 2.2. The Cornea

The cornea is a clear, avascular structure through which light enters the eye. Lining the inner surface of the stroma is the corneal endothelium. sAC was first identified in the endothelial layer of cornea by Sun et al. in 2003 [32].

The corneal endothelium allows nutrients from the aqueous humour into the stroma, while also transporting H_2_O out to maintain dehydration of the stroma [37]. This process is stimulated by bicarbonate and follows a Cl^−^ gradient [37]. An increase in cAMP activity results in higher net endothelial fluid transport [38]. Li et al. investigated the role of sAC in apoptosis in corneal endothelial cells (CECs) and found that bicarbonate-dependent endogenous sAC activity can initiate antiapoptotic signal transduction, suggesting a role for sAC in cellular protection [39]. 

The corneal endothelium does not undergo mitosis in vivo; therefore, it relies on a large functional reserve of cells. CEC density decreases by approximately 0.5% per year in healthy individuals; however, in those with endothelial dystrophies or following surgery or trauma, the loss of CEC can be devastating for maintaining dehydration of the stroma, leading to reduction in vision [39]. As it is suggested that AC modulation may protect CEC against stress [40], this could be an area of therapeutic interest.

The cAMP-dependent activation of the cystic fibrosis transmembrane conductance regulator (CFTR) regulates fluid transport in many tissues, including CEC, through chloride and bicarbonate ion transport [32]. These processes are essential for maintaining fluid balance and cellular homeostasis. In the corneal endothelium, proper fluid transport is crucial for maintaining corneal transparency and hydration. In cystic fibrosis (CF), defective CFTR leads to altered ion transport and fluid balance in corneal endothelial cells (CECs). This can affect the regulation of intraocular pressure and the corneal dehydration process. It has been demonstrated that bicarbonate-stimulated sAC contributes to baseline cAMP in bovine corneal endothelial cells, therefore affecting CFTR activity [21]. In most cases, patients with CF do not have visual problems. It has, however, been shown that in CF, there is a significantly higher density of CECs compared to normal, which may be the evidence of compensation for the CFTR defect [41].

### 2.3. The Crystalline Lens

Ion and water balance are essential for preserving lens transparency. Disruptions in this balance can lead to cataracts, characterized by lens opacity and impaired vision [42]. Understanding the role of ACs and calcium signalling in the lens can provide insights into the mechanisms that maintain lens clarity and function, potentially leading to new treatments for cataract prevention and other lens-related disorders.

The lens epithelium is pivotal in regulating the homeostatic functions of the lens, marking it as a region of high metabolic activity associated with adenylyl cyclases (ACs) [43,44,45]. As in the cornea, proper ion and water homeostasis is required to maintain lens transparency and normal vision.

The lens’s response to hypoosmotic stress involves the activation of transient receptor potential cation channel subfamily 4 (TRPV4). This channel facilitates the entry of calcium into the cytoplasm, subsequently activating the calcium-sensitive isoforms of ACs. This mechanism was first demonstrated in porcine lenses by Shahidullah et al. in 2017 [46].

### 2.4. The Ciliary Body

The ciliary body is anatomically situated between the scleral spur and retina. It not only mediates the optical accommodation of the crystalline lens, but also produces aqueous humour (AH) via ultrafiltration [47]. Relaxation of the ciliary muscle in cats has been found to be mediated by both cAMP-dependent and independent mechanisms [48].

AH is produced by pigmented epithelial cells and non-pigmented epithelial cells found in the ciliary processes. This fluid provides nutrients to the avascular tissues in the cornea and lens. AH is continually produced and drained from the eye, exhibiting circadian rhythm which has been found to be governed by AC in rabbit ciliary processes [49]. This process generates intraocular pressure (IOP) through the resistance to drainage of AH. 

IOP is essential for maintaining the optical properties and shape of the eye [50]. A rise in IOP causes the death of RGCs; therefore, the treatment of glaucoma aims to lower IOP. Thus, this pathway has been of great interest for developing new ways of preventing vision loss in such a prevalent disease. 

cAMP and bicarbonate have established roles in regulating IOP [51,52,53]. cAMP is understood to facilitate drainage by the conventional outflow pathway, which is formed by the trabecular meshwork (TM) and Schlemm’s canal [54,55,56,57,58,59,60]. Flow through the TM is governed by many molecules, including prostaglandins (analogues of which are a commonly used therapy in treating glaucoma), which then stimulates AC activity in a dose-dependent fashion [61,62]. A comprehensive review paper on the role of cAMP in glaucoma was produced by Shim et al. in 2017, highlighting RGC regeneration by sAC, as well as optic nerve head astrocyte restoration by tmAC [63]. 

Bicarbonate sensitive AC was identified in the rabbit ciliary body in 1993 by Mittag et al., and work began to identify molecules that could influence this activity, such as beta-adrenergic agonists [14]. In 2014, Lee et al. described how sAC plays a significant role in the regulation of outflow resistance through their work on mice, noting increases in IOP when an sAC was inhibited [47]. Further characterisation of this pathway, and therefore the regulation of outflow, could potentially provide an area of novel glaucoma therapy.

Acetazolamide is a carbonic anhydrase inhibitor; therefore, it reduces levels of bicarbonate. It is commonly used in treating high IOP. Shahidullah et al. demonstrated how the exposure of acetazolamide to porcine non-pigmented ciliary epithelium increased cAMP in a response that involved the activation of sAC [64]. However, in 2023, Wiggins et al. examined the effect on IOP from brinzolamide (a clinically used carbonic anhydrase inhibitor) and found that sAC knockout, wild-type and wild-type mice treated with sAC inhibitors all showed a significant decrease in IOP, thereby suggesting that the signalling cascade by which brinzolamide regulates IOP does not involve sAC in mice [65]. Further research to elucidate the exact pharmacodynamics could lead to better medicine management and patient-targeted therapies. A review paper by Agarwal et al. in 2014 discusses new targets of the modulation of IOP, with a focus on adenosine receptor signalling pathways, including the activation of AC [66].

### 2.5. The Retina

The retina is composed of photoreceptive cells and layers of neurons and glia. cAMP and AC are understood to play a role in many aspects of vision, including neuritic sprouting [67,68], dopamine (DA) release [69] and the generation of the retinal response to light [70]. Most research on sAC and AC in the retina has focussed on its function in retinal ganglion cells (RGCs) [18,27,71,72] and photoreceptors [73]. 

RGCs are the neurons that transmit visual information from photoreceptors via bipolar and retina amacrine cells to the brain, with their axons comprising the optic nerve. Most RGCs synapse in the lateral geniculate nucleus. The remaining RCGs involved in pupillary reflexes and circadian functions terminate in other areas of the brain [74]. Their death causes the irreversible vision loss in glaucoma. 

In 2009, Dunn et al. first reported a potential role of sAC in Ca^2+^-dependent protein kinase A (PKA) signalling [27]. It was found that the signalling pathway still functioned despite the blockade of tmAC, suggesting sAC was responsible for PKA activation. Building on the work of Varella et al., who identified the role of PKA in apoptosis in new-born rat retina [75], other molecules such as the pituitary adenylate cyclase-activating polypetide (PACAP) have been shown to provide neuroprotection through anti-apoptotic effects [76,77,78].

In the developing retina, increased intracellular levels of cAMP protect cells from degeneration [79,80]. Corredor et al. studied whether sAC-generated increases in cAMP had an effect on axon growth in RGCs. Bicarbonate was applied to cultured RGCs, where it was found to increase growth but have no impact on cell survival, unlike electrical stimulation. In contrast, electrical stimulation did have an effect on survival [18]. Notably, tmAC inhibitors had no effect on axon survival or growth in mice, which was in line with work by Dunn et al. who suggested tmACs may be less significant than sAC in this respect [27]. The importance of this research lies in its contribution to understanding the signal transduction pathways that govern neuron survival and the potential role they could play in developing therapies for neurodegenerative diseases and central nervous system injuries.

A recent paper by Cameron et al. described the impact sAC and cAMP have on the formation of neuroprotective astrocytes, which subsequently inhibit neurotoxic astrocytes to promote RGC survival [81] and suggest that sAC may be vital in the treatment of glaucoma and other optic neuropathies. Their work also developed a new viral vector to target optic nerve astrocytes, representing an important step in progressing gliotherapeutic approaches.

The reduced form of vitamin C, ascorbate, is highly concentrated in the central nervous system (CNS), including the retina. DA, through D_1_- and D_2_-like receptor subfamilies, are classically coupled to AC. These interactions are known to modulate synaptic transmission in the retina. A study by de Encarnação et al. using primary retinal cultures showed that DA plays a crucial role in regulating ascorbate homeostasis through a signalling pathway involving D_1_R/AC/cAMP/ exchange protein directly activated by cAMP type 2 (EPAC2), suggesting that vitamin C may modify DA neurotransmission in the retina [82]. 

### 2.6. Retinal Disease

Retinopathy of prematurity (ROP) is an ocular disease that can occur in premature babies, before retinal vessels complete their normal growth [83]. The upregulation of the vascular endothelial growth factor (VEGF) through high oxygen at birth, fluctuations in oxygenation, nutrition and poor postnatal growth causes aberrant vessel formation, which can ultimately lead to retinal detachment. An observational case–control-targeted genetic analysis by Paradis et al. found adenylyl cyclase 4 (ADCY4), ADCY7 and ADCY 9 genes (and others) were associated with ROP [83]. The multicentre validation of these newly discovered risk factors could help develop tools for predicting and preventing the development of severe ROP. 

Stargardt disease is a severe juvenile form of macular degeneration [84]. It is associated with more than 800 mutations in the ABCA4 gene, an ATP-binding cassette transporter that moves all-trans-retinal from the internal membranes of retinal outer segment discs to the cytoplasm, where it is reduced by retinol dehydrogenases to retinol. Detailed analysis of AC in the context of Stargardt disease by Chen et al. in 2013 found that pharmacological interventions targeted at both G-protein-coupled receptor signalling pathways and AC improved photoreceptor cell survival, attenuated the pathological deposits in the retina and persevered photoreceptor function [84].

### 2.7. Retinal Circadian Rhythm

Photoreceptor metabolic activity varies significantly in dark versus light, causing changes in CO_2_/bicarbonate concentration in the outer retina [85]. A study on cell lines derived from human retinal pigment epithelium by Pavan et al. in 2006 demonstrated a circadian rhythm in the expression of AC and clock genes, which could then represent relevant drug targets for diseases involving circadian dysfunctions [31]. 

Research on chicken embryo retina found that the activation of AMP-activated protein kinase A (AMPK), downstream of AC, is under circadian control, and anti-phase to the retinal ATP rhythm [86,87,88]. Further research by Hwang et al. investigated the circadian rhythm of contrast sensitivity in RGCs [89]. Spatial variation in light intensity (known as spatial contrast) makes up the majority of the visual information perceived by mammals. Contrast sensitivity is the relative ability to detect contrast [90]. Retinal dopamine receptors (D4Rs) have been implicated in this process, functioning alongside a clock-controlled AC gene ADCY1. Contrast sensitivity was found to be reduced in mice lacking ADCY1 [89,91].

Melatonin is synthesized by photoreceptors at high levels at night and lower levels during the day. It exercises its influence by interacting with a family of G-protein-coupled receptors that are negatively coupled with AC. Fukuhara et al. reported that circadian control of the cAMP-signalling cascade was linked to the rhythmic control of type 1 adenylyl cyclase (AC1) expression [92]. Research in the field of exogenous melatonin as a benefit to ocular health is not extensive, but it has certainly been linked to retinal physiology and pathophysiology [93].

Disruption to the circadian rhythm can have grave effects on the physiological functioning of the body. The research into the role of AC within this area may, therefore, have implications for a huge number of homeostatic pathways and their dysfunction.

### 2.8. The Lacrimal Gland

Dry eye is the most common ocular surface disease. In large epidemiological studies, the age-specific prevalence ranges from 5 to 30% [94]. Currently, there are only limited treatment options, resulting in significant management challenges. Tear secretion is an intricate process which involves main and accessory lacrimal glands (LGs), oil-producing Meibomian glands and corneal and conjunctival epithelial goblet cells, which yield mucus. LG dysfunction results in aqueous deficient dry eye. 

Vasoactive intestinal peptide (VIP), released by parasympathetic nerves, is an important regulator of lacrimal gland function [95]. It is also a smooth muscle relaxant and vasodilator peptide in the lung [96]. VIP was implicated in the regulation of tear production in the case of a patient with a VIP-secreting metastatic pancreatic adenocarcinoma, who had serum levels 80 times higher than normal and significant tear overproduction [97]. 

The effect of VIP on ductal fluid secretion was studied by Berczeli et al. in CFTR-knockout mice and wildtypes, with ductal fluid secretion measured with videomicroscopy [30]. In this study, VIP stimulation resulted in a continuous fluid secretion from wildtype mice, as opposed to weak pulse-like secretions from the CFTR-knockout mice. A small (but statistically significant) increase was detected in the intracellular Ca^2+^ level, which implicated the role of the AC-cAMP-CFTR route in this function [30]. LG secretion is mediated by a wide range of ion transporters and channels. This study suggests that the modification of CFTR function may be a target to stimulate LG secretion, and therefore, a possible option in treating aqueous deficient dry eye.

## 3. Conclusions

The exploration of adenylyl cyclases (ACs) across various eye structures offers invaluable insights into their multifaceted roles and potential therapeutic avenues. Pioneering efforts, such as Corredor et al.’s utilization of viral vectors to target sAC in optic nerve astrocytes, signify a pivotal advancement toward practical therapeutic applications [18].

Vision-threatening conditions like corneal trauma, endothelial disease, and glaucoma pose significant challenges. Targeting sAC offers promising avenues for novel treatments by regulating corneal stromal hydration, enhancing corneal endothelial cell survival, and modulating aqueous humour outflow from the ciliary body. Furthermore, the intersection of research on brain injuries, glial survival, and glaucoma underscores the immense value of knowledge sharing across diverse medical domains.

The intricate interplay between sAC, cyclic adenosine monophosphate (cAMP), and the cystic fibrosis transmembrane conductance regulator (CFTR) highlights the potential for broader applications beyond their initial contexts. Notably, the circadian rhythm of AC expression and activity suggests their involvement in regulating ocular circadian functions, presenting novel targets for circadian rhythm disorders.

There are some limitations in the studies covered in this systematic review. ACs are widely found within cell-signalling cascades, which means precise targeting may be a challenge for future therapies. Furthermore, some of the research has been carried out on animal models, which makes assumptions in their relevance to human physiology.

In conclusion, the multifaceted roles of ACs in ocular physiology and pathology position them as promising targets for continued research and the development of innovative therapeutic interventions in ophthalmology and related disciplines, paving the way for enhanced patient care and improved visual outcomes.

## Data Availability

No new data were created or analysed in this study. Data sharing is not applicable to this article.

## Abbreviations

AC	Adenylyl cyclase
AH	Aqueous humour
AMPK	Adenosine monophosphate activated protein kinase A
ATP	Adenosine-5′-triphosphate
CA	Carbonic anhydrase
cAMP	Cyclic adenosine 3′,5′ monophosphate
CAP1	Adenylyl-cyclase-associated protein 1
CEC	Corneal endothelial cell
CFTR	Cystic fibrosis transmembrane conductance regulator
CNS	Central nervous system
D4R	Retinal dopamine receptor
DA	Dopamine
GPCRs	G-protein coupled receptors
IOP	Intraocular pressure
LG	Lacrimal gland
MS	Multiple sclerosis
PACAP	Pituitary adenylate cyclase-activating polypeptide
PKA	Protein kinase A
RGCs	Retinal ganglion cell
ROP	Retinopathy of prematurity
sAC	Soluble adenylyl cyclase
sAC_fl_	Full-length mammalian Soluble adenylyl cyclase
TM	Trabecular meshwork
TRPV4	Transient receptor potential cation channel subfamily 4
tmAC	Transmembrane adenylyl cyclase
VEGF	Vascular endothelial growth factor
VIP	Vasoactive intestinal peptide
